# Laminin-5 offsets the efficacy of gefitinib (‘Iressa’) in hepatocellular carcinoma cells

**DOI:** 10.1038/sj.bjc.6602231

**Published:** 2004-11-16

**Authors:** G Giannelli, A Azzariti, E Fransvea, L Porcelli, S Antonaci, A Paradiso

**Affiliations:** 1 Section of Internal Medicine, Department of Internal Medicine, Immunology, and Infectious Diseases, University of Bari Medical School, Bari, Italy; 2 Clinical Experimental Oncology Laboratory, National Cancer Institute Bari, Italy

**Keywords:** Laminin-5, HCC, gefitinib, extracellular matrix proteins, tumour growth

## Abstract

Prognosis and survival of patients with hepatocellular carcinoma (HCC) is still very poor, and no therapies are currently available to inhibit tumour growth and metastases. Recently, we reported that the expression of an extracellular matrix component (ECM), namely Laminin-5 (Ln-5), is directly related to poor prognosis in HCC patients. The aim of our study is to investigate the preclinical effect of gefitinib in an *in vitro* HCC model. We found that the IC_50_ of gefitinib in HCC cells ranged from 0.7 to 10.0 *μ*M, whereas Ln-5 inhibited the activity of gefitinib in a dose-dependent manner. Complete inhibition of phosphorylated (p)-EGFR (epidermal growth factor receptor) was obtained within 6 h exposure to gefitinib and complete restoration of the receptor status was obtained after 24 h. A downstream effect yields a decrease in p-Akt and p-Erk 1/2. The addition of exogenous Ln-5 has no effect on p-EGFR, whereas it restores p-Erk 1/2 and p-Akt. Consistently, Ln-5 induces recovery of HCC cells from Gefitinib-induced apoptosis. In conclusion, gefitinib inhibits HCC cell growth and we report for the first time that Ln-5, but not other ECM molecules, reduces the ability of gefitinib to inhibit cell growth via Akt. As patients with HCC have different Ln-5 expression levels, these results may help to better understand which patients might benefit from gefitinib treatment.

Hepatocellular carcinoma (HCC) is the fifth most frequent malignancy in the world and the third cause of tumour-related death. Furthermore, it is estimated that in coming years, the occurrence of HCC will increase in Western countries, due to hepatitis C virus diffusion (Bruix and Llovet, 2002). In HCC patients, prognosis and survival are still unsatisfactory mainly because of tumour recurrence and metastatic diffusion (Giannelli *et al*, 2002b).

The molecular mechanisms responsible for HCC spread are not yet known; however, we have recently reported that the expression of an extracellular matrix (ECM) component, namely Laminin-5 (Ln-5), is strongly associated *in vivo* with a more aggressive and invasive phenotype of HCC and with a worse prognosis (Giannelli *et al*, 2003). Ln-5, a major component of the basement membrane, plays a crucial role for epithelial cells so that its genetic absence is responsible for lethal disease (Meneguzzi *et al*, 1992). It promotes a number of different biological functions including adhesion, migration and scattering and it has been reported to play a role in cancer metastasis (Miyazaki *et al*, 1993; Baker *et al*, 1996; Xia *et al*, 1996). Integrins *α*3*β*1 and *α*6*β*4, the two main receptors for Ln-5, are highly expressed in epithelial cancer cells (reviewed in Giannelli *et al*, 2002a).

So far, no drugs are available to inhibit tumour growth and/or metastatic spread of HCC and current therapies are restricted to surgery, ethanol injection, radiotherapy and chemoembolisation. Recently, gefitinib (‘Iressa’), an orally active epidermal growth factor receptor (EGFR) tyrosine kinase inhibitor that blocks signal pathways implicated in cancer growth and metastasis, has been successfully used in lung and colon cancer (Douglass, 2003; Haringhuizen *et al*, 2004; Johnson *et al*, 2004), where it could represent an alternative monotherapy for patients with advanced tumours (Herbst, 2003). In two large-scale Phase II trials, approximately 50% of previously treated patients with NSCLC had either an objective response or disease stabilisation following treatment with gefitinib 250 mg day^−1^ (Fukuoka *et al*, 2003; Kris *et al*, 2003). Furthermore, it has been reported to reduce tumour growth and metastasis occurrence in a xenograft HCC animal model (Matsuo *et al*, 2003), and in the USA a Phase II trial treating patients with HCC with gefitinib is currently underway.

The goal of our study is to investigate the potential therapeutic effect of, and the potential resistance to, gefitinib in an *in vitro* HCC model; in particular, we analysed gefitinib's ability to inhibit cell growth, and whether the Ln-5 ECM molecule reduced its efficacy.

## MATERIALS AND METHODS

Gefitinib activity was tested on four different human HCC cell lines, in the absence and presence of Ln-5.

### Reagents

Gefitinib was kindly provided by AstraZeneca Pharmaceuticals, UK. Stock solutions were prepared at 20 mM in dimethyl sulphoxide (DMSO) and stored in aliquots at −20°C. Purified Ln-5 was prepared as previously described (Koshikawa *et al*, 2000). Working dilutions were made in culture medium supplemented with 10% fetal bovine serum, 2 mM glutamine, 50 000 UL^−1^ penicillin and 80 *μ*M streptomycin.

Laminin-1 (Ln-1) and Collagen IV (Coll IV) were purchased from Sigma Chemical Company (St Louis, MI, USA), Coll I from BD Biosciences (Bedford, MA, USA), Fibronectin (Fn) from Calbiochem (La Jolla, CA, USA), Fibrinogen (Fg) and Vitronectin (Vn) were gifted by Dr Felding-Habermann (TSRI, La Jolla, CA, USA) (Felding-Habermann *et al*, 2002).

### Cell culture

Alexander, HepG2, HLF and Sk-Hep1 human HCC cell lines were routinely cultured in RPMI or DMEM medium supplemented with 10% fetal bovine serum (2 mM glutamine, 50 000 UL^−1^ penicillin and 80 *μ*M streptomycin) in a humidified incubator at 37°C with an atmosphere containing 5% CO_2_. Cells were trypsinised once a week with trypsin/ethylenediaminetetraacetic acid (EDTA) (0.25%/0.02%) and the medium was changed twice a week (Giannelli *et al*, 2001).

### Antibodies

Antiphosphotyrosine polyclonal antibody PY99 was purchased from Santa Cruz Biotechnology, USA; anti-EGFR monoclonal antibody from Becton-Dickinson (San Diego, CA, USA) and the following monoclonal antibodies were from Cell Signaling (USA): anti-Akt, anti-phosphoAKT, anti-ERK1/2 and anti-phospho-ERK1/2. Mouse and rabbit horseradish peroxidase (Amersham Pharmacia Biotech, Upsala, Sweden) were used as secondary antibodies.

Anti-*α*3 and anti-*α*6 integrin blocking antibodies were purchased from Gibco (Gaithersburg, MA, USA) and from Pharmingen (San Diego, CA, USA).

### Cytotoxicity assay

Gefitinib determination of IC_50_ was performed using the 3-[4,5-dimethylthiazol-2-yl]-2,5-diphenyltetrazoliumbromide (MTT) assay. Briefly, 7500 cells in a volume of 200 *μ*l were plated in each well on a 96-well plate. After 24 h, cells were challenged for 3 days with gefitinib at the following concentrations: 0.01, 0.1, 0.5, 1.0, 5.0, 10.0 and 50.0 *μ*M. Each experiment was reproduced in seven different wells, and each experiment repeated at least three times. Results were expressed as a dose–response curve with a plot of the fraction of unaffected (surviving) cells *vs* the drug concentration. IC_50_ was defined as the drug concentration yielding 50% of affected (nonsurviving) cells compared with untreated controls. In some experiments, Ln-1, Ln-5, Coll IV, Coll I, Fg, Fn and Vn (1 *μ*g ml^−1^) were added to cells in the presence of gefitinib IC_50_ and the cell growth inhibition was measured by the MTT assay. Ln-5 was also tested at different concentrations (ranging from 0.3 up to 3.0 *μ*g ml^−1^).

Anti-*α*3 and anti-*α*6 integrin-blocking antibodies (30 *μ*g ml^−1^) were added together with Ln-5 in the presence of gefitinib IC_50_ and the cell growth inhibition was measured by the MTT assay.

### Western blot analysis

Protein samples were extracted from gefitinib (IC_50_)- and/or Ln-5 (1 *μ*g ml^−1^)-treated cells (3 × 10^6^ cells) after homogenisation in RIPA buffer (0.5 M NaCl, 1% Triton X-100, 0.5% NP40, 1% deoxycolic acid, 3.5 mM sodium dodecyl sulphate (SDS), 8.3 mM Tris HCl pH 7.4, 1.6 mM Tris base) and treated with a 20% protease inhibitor cocktail (Sigma, MO, USA). Protein concentration was determined by the Bradford method, samples were normalised for protein concentration (25–50 *μ*g), and electrophorised on 10–12.5% SDS–PAGE. The signal was detected by the chemoluminescence assay (ECL-Plus, Amersham Life Science, UK) and the expression level was calculated by densitometric analysis using Multi-Analyst software (Biorad, Hercules, CA, USA), using *β*-actin expression as internal standard.

### Immunoprecipitation analysis

HCC cells (3 × 10^6^) were treated with gefitinib (IC_50_) and/or Ln-5 (1 *μ*g ml^−1^) for 24 h. Cells were lysed in RIPA-IP buffer (140 mM NaCl, 20 mM Tris/HCl, 10 mM EDTA pH 8, 10% glycerol, 1% NP40, 1 mM Na-deoxycolic acid, 1 mM phenylmethylsulphonyl fluoride (PMSF)), passed through a 22-gauge syringe and cleared by centrifugation at 10 000 **g** at 4°C for 10 min. Proteins were immunoprecipitated by incubating 0.1–0.3 mg of total cell lysate with 0.2 *μ*g of antiphosphotyrosine antibody or anti-EGFR antibody for 1 h at 4°C. In all, 2–5 *μ*l of protein A/G agarose (Santa Cruz Biotechnology, USA) were incubated overnight at 4°C. Cell suspension was centrifuged at 2600 r.p.m. and the pellet was washed three times with phosphate-buffered saline (PBS) and then resuspended in 10 *μ*l of Laemmli buffer. Each sample was separated on 10% acrylamide gel and Western blot was performed as described above.

### Apoptosis analysis

Apoptosis detection was performed by Annexin V-FITC staining assays (Biovision, Palo Alto, CA, USA). In accordance with the manufacturer's instructions, HCC cells (5 × 10^5^) were incubated with Annexin V at room temperature for 10 min. Apoptotic cells were detected by FACS analysis (Becton-Dickinson, NJ, USA) and quantified using cellquest software (Becton-Dickinson).

Apoptosis detection was further investigated by the Cell Death ELISA^PLUS^ kit (Roche Molecular Biochemicals, Milan, Italy). The test is based on the detection of mono- and oligonucleosomes in the cytoplasmic fraction of cell lysates by biotinylated antihistone-coupled antibodies, and their enrichment in the cytoplasm is calculated as the absorbance of sample cells/absorbance of control cells. The enrichment factor was used as a parameter of apoptosis and shown on the *Y*-axis as mean±standard deviation (s.d.). Experiments were performed according to the manufacturer's instructions.

### Statistical analysis

Results are expressed as the mean±s.d. and the statistical significance of the Ln-5-dependent reduction of gefitinib activity was determined by Student's *t*-test with a 95% confidence interval.

## RESULTS

All the experiments of gefitinib-induced cytotoxicity were evaluated after 3 days of continuous exposure, therefore the use of FCS in the *in vitro* culture system was required to ensure cell vitality; this is consistent with other studies (Magne *et al*, 2003; Tortora *et al*, 2003; Xu *et al*, 2003b). Furthermore, we have previously shown the stability of the drug in the presence of serum in the HPLC system (Porcelli *et al*, 2004).

### Epidermal growth factor receptor and downstream effectors expression on HCC cell lines

**Figure 1:**
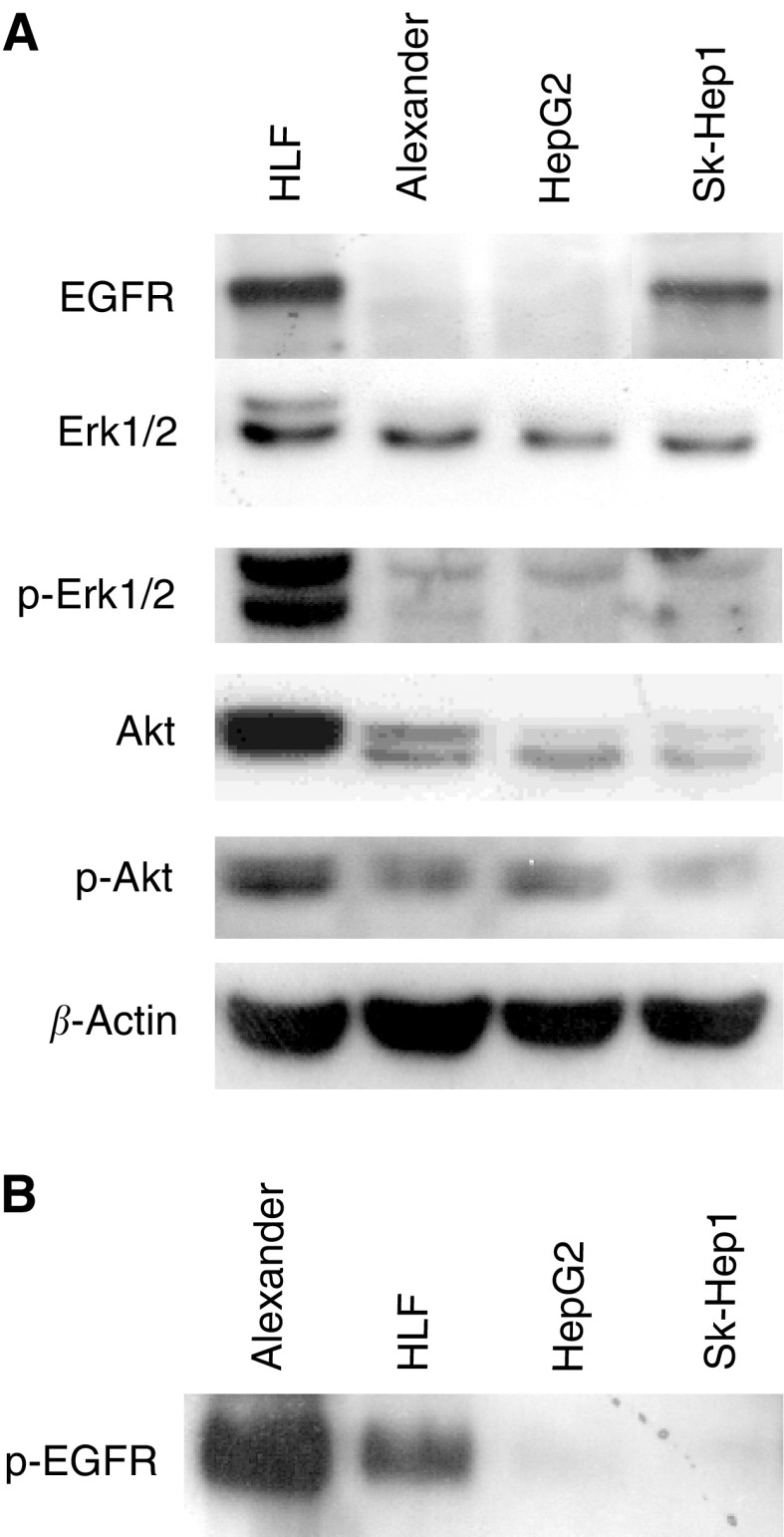
EGFR and downstream effectors expression on HCC cell lines. EGFR, Erk 1/2, p-Erk 1/2, Akt, p-Akt expression in HLF, Alexander, HepG2 and Sk-Hep1 cellular extracts were analysed by Western blotting (**A**). Phosphorylated-EGFR was determined by immunoprecipitation followed by Western blotting (**B**).

As shown in Figure 1

, the expression level of EGFR is higher in HLF and Sk-Hep1 cells than in Alexander and HepG2 cells, while the p-EGFR form is more evident in Alexander and HLF cells than in HepG2 and Sk-Hep1 cells. On the contrary, no differences were observed between HCC cell lines in terms of Erk 1/2, p- Erk 1/2, Akt and p-Akt expression.

### Gefitinib inhibits HCC cell lines growth

To provide preclinical evidence of the effectiveness of gefitinib in HCC, we tested the sensitivity of four distinct human HCC cell lines to different drug concentrations after 3 days incubation, consistent with a long period of drug utilisation in clinical trials.

All the cell lines were investigated under the same experimental conditions to limit biological variability. As reported in Table 1

**Table 1:** Gefitinib IC_50_ in HCC cell lines after 3 days incubation

**Cell lines**	**Gefitinib (*μ*M)**
Alexander	0.67±0.22
HepG2	4.73±0.42
HLF	4.04±0.54
Sk-Hep1	10.1±1.76

, HCC cell lines showed similar gefitinib IC_50_ values_._ Experiments were repeated at least three times, and the mean and standard deviation refer to all the experiments.

### Extracellular matrix proteins modulate gefitinib effectiveness

To investigate whether ECM proteins such as Ln-1, Ln-5, Coll I, Coll IV, Fg, Fn and Vn can interfere with gefitinib's ability to inhibit cell growth of HCC cell lines, we added exogenous matrices (1 *μ*g ml^−1^) to the cells in the presence of gefitinib IC_50_. As shown in Table 2

**Table 2:** Percentage of survived HCC cells treated with Gefitinib (IC_50_) in the presence of ECM proteins

	**Sk-Hep1**	**Alexander**	**HLF**	**HepG2**
Gefitinib	50±3.7	50±1.3	50±8.4	50±10
Gefitinib+LN-5	78±6.9	84±11.0	81±5.8	81±09
Gefitinib+Coll I	62±6.9	55±7.9	56±9.5	58±6.8
Gefitinib+Coll IV	63±7.0	57±2.3	58±9.2	61±5.9
Gefitinib+Fib	61±6.6	55±4.8	57±6.2	59±7.9
Gefitinib+FN	62±5.4	57±1.8	55±8.3	59±6.9
Gefitinib+LN-1	57±9.7	54±2.7	59±5.0	55±4.7
Gefitinib+VN	59±7.5	58±1.0	56±5.6	57±6.8

Each experiment was carried out in triplicate.

, gefitinib-dependent cell cytotoxicity (50%) was dramatically reduced by the presence of Ln-5, with a consequent significant increase of cell survival (*P*<0.001), whereas no effect was observed with any of the other ECM proteins used. However, this Ln-5 activity was completely inhibited by anti-*α*3 but not by anti-*α*6 integrin-blocking antibodies in HLF cells, and was completely inhibited by anti-*α*6 in Alexander cells. These results are consistent with our previous work reporting a strong expression of *α*3 integrin in HLF, while Alexander cells were *α*3 integrin negative but intensely *α*6 positive (Giannelli *et al*, 2001).

**Figure 2:**
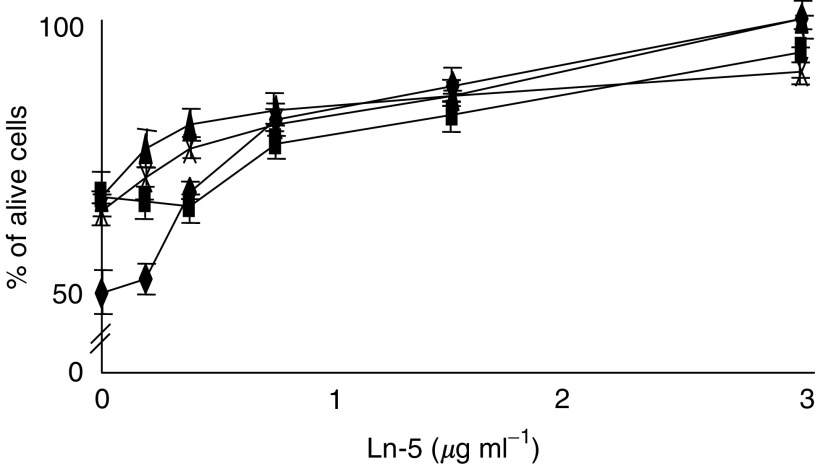
Ln-5 modulation of Gefitinib IC_50_ effectiveness on HCC cells is dose-dependent. Ln-5 inhibits Gefitinib IC_50_ effectiveness at 3 *μ*g ml^−1^, while it is not effective at 0.03 *μ*g ml^−1^ on HLF (♦), Alexander (▪), Sk-Hep1 (▴) and HepG2 (X) cells.

To better characterise the interaction of Ln-5 with gefitinib treatment, HCC cell lines were incubated with gefitinib (IC_50_) in the presence of different Ln-5 concentrations. As shown in Figure 2

, gefitinib's ability to inhibit cell growth was Ln-5 dose-dependent; in particular, complete inhibition of the drug-induced effect was obtained with 3 *μ*g ml^−1^ of Ln-5, while no changes were observed with 0.03 *μ*g ml^−1^ of Ln-5.

In conclusion, gefitinib inhibited growth of HCC cell lines, whereas Ln-5 inhibited the gefitinib-induced activity in a dose-dependent manner.

### Cellular response to gefitinib and/or Ln-5

#### Molecular targets

To study the molecular mechanisms responsible for the interaction between gefitinib and Ln-5, we analysed the modulation of drug-specific cell targets such as EGFR, as well as the main steps of its signal transduction pathways, including Erk1/2 and Akt.

Neither gefitinib nor Ln-5 affected EGFR, Akt and Erk1/2 expression compared to controls using *β*-actin expression as internal standard.

**Figure 3:**
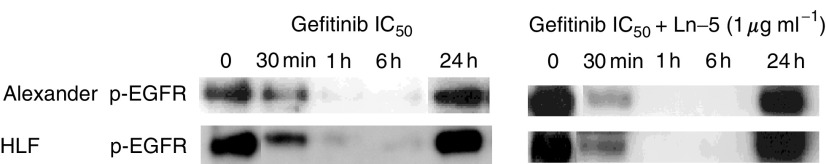
Time course of Gefitinib-dependent p-EGFR inhibition in HCC cells in the presence and absence of Ln-5. p-EGFR is completely inhibited after 6 h and completely recovers after 24 h both in the presence and absence of Ln-5.

**Figure 4:**
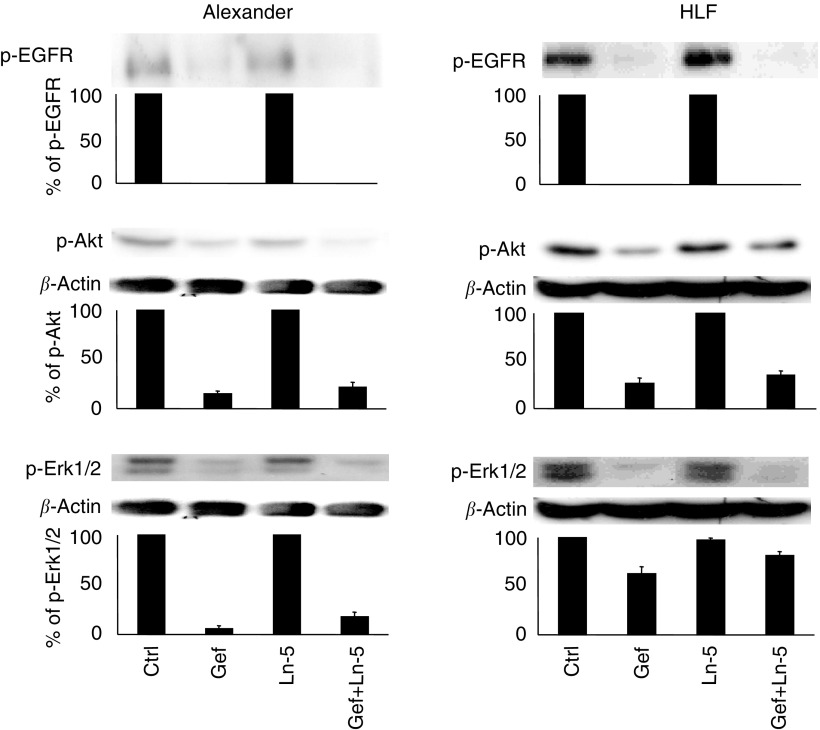
Ln-5 modulation of 6 h Gefitinib activity on molecular targets. Ln-5 (1 *μ*g ml^−1^) does not reverse Gefitinib (IC_50_) effectiveness on p-EGFR, but partially restores p-Akt and p-Erk 1/2 on Alexander and HLF cells.

On the contrary, the p-EGFR form was completely inhibited after 6 h of drug treatment (IC_50_) and recovered after 1 day, while no effect was observed after the addition of Ln-5 (Figure 3

). Consistently, p-Erk 1/2 and p-Akt were both inhibited, with some differences between Alexander and HLF cells. After a short time of drug exposure (6 h), gefitinib inhibited both effectors more efficiently in Alexander than in HLF cells (Figure 4

). No effect of Ln-5 alone was observed on either effector, whereas Ln-5-treated cells showed a reduced gefitinib inhibition of p-Akt from 15 to 25% (*P*>0.05) and from 25 to 35% (*P*>0.05) in Alexander and HLF cells, respectively, and of p-Erk1/2 from 2 to 20% (*P*=0.02) and from 60 to 80% (*P*=0.03) in Alexander and HLF cells, respectively.

**Figure 5:**
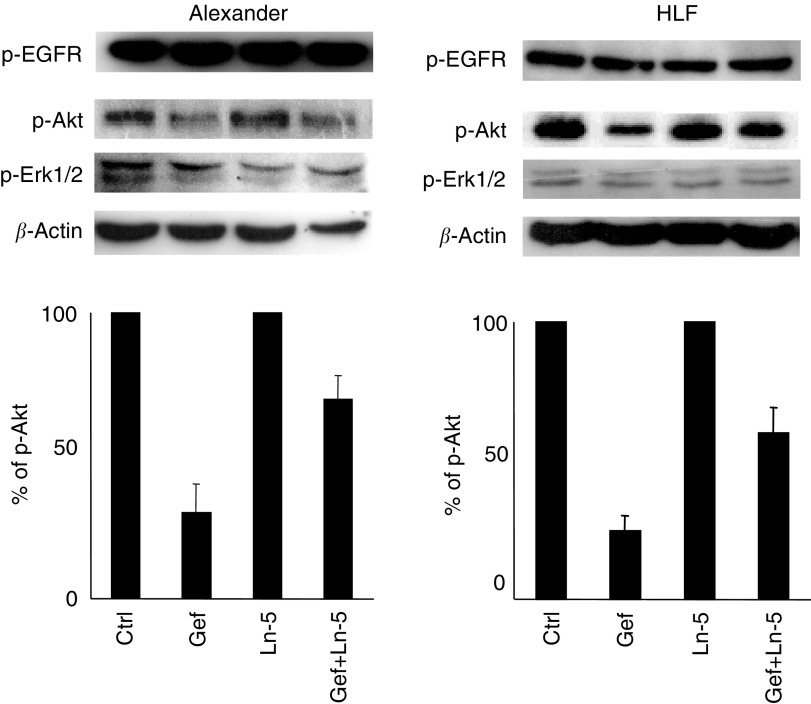
Ln-5 modulation of 24 h Gefitinib activity on p-Akt. Gefitinib (IC_50_) does not affect p-EGFR and p-Erk 1/2 on Alexander and HLF cells. With Ln-5 (1 *μ*g ml^−1^), p-Akt recovers (40%) in both cell lines.

After 24 h of gefitinib and/or Ln-5 exposure, no differences between Alexander and HLF cells were evident. As shown in Figure 5

, p-Erk1/2 returned to baseline even in the absence of Ln-5, whereas p-Akt inhibition decreased, in the presence of Ln-5, from 70 to 30% (*P*<0.02) and from 60 to 20% (*P*<0.02) in Alexander and HLF cells, respectively. In conclusion, the recover of the effector forms in the presence of Ln-5 is time-dependent.

### Apoptosis induction

Apoptotic cells were quantified using Annexin V and cytoplasmic histone-associated-DNA fragmentation assays. After three days of gefitinib exposure, there were 7 and 5% Annexin V positive cells and the mono- and oligonucleosomes enrichment factor was 4.3±0.4 and 3.8±0.2, in Alexander and HLF cells, respectively. Consistently with the Ln-5 effect on gefitinib-dependent p-Akt inhibition, in Ln-5-treated cells exposed to gefitinib treatment, Annexin-positive cells were less than 1% while the enrichment factor was less than 1.8±0.2 in both cell lines.

## DISCUSSION

Prognosis and survival of HCC patients is still poor. Nevertheless, no drug treatment is currently available to block or reduce cancer growth and/or tumour metastasis. This is mainly because the molecular mechanisms regulating the aggressive and malignant phenotype of HCC are still unknown. However, it has been reported that the expression of Ln-5 is strongly correlated with a worse prognosis in HCC patients (Giannelli *et al*, 2003). No therapies are so far available to inhibit or reduce HCC growth and invasion.

Gefitinib, a target-oriented drug that inhibits EGFR tyrosine kinase, has been successfully used to reduce lung and colon cancer growth and metastasis (Sirotnak, 2003; Xu *et al*, 2003a, 2003b; Azzariti *et al*, 2004). Recently, it has been reported that gefitinib reduces tumour growth and metastatic spread of HCC in a mouse experimental model (Matsuo *et al*, 2003). Furthermore, a Phase II trial with gefitinib in patients with HCC is currently ongoing in the USA; however, no data are yet available on HCC cell sensitivity.

In this study, we investigated the effectiveness of an *in vitro* HCC model to gefitinib. Our results show that gefitinib is effective on HCC growth, but its activity is inhibited by Ln-5. We base this conclusion on the following data: (1) gefitinib inhibits HCC cell growth; (2) Ln-5 antagonises gefitinib growth inhibition in a dose-dependent manner; (3) gefitinib induces persistent dephosphorylation of Akt, while with Ln-5 the p-Akt form recovers; (4) gefitinib-induced late apoptosis is reversed by Ln-5.

In other cancers, such as colon and lung carcinoma, the downstream effect of inhibiting EGFR phosphorylation involves both the Akt and Erk1/2 pathways (Azzariti *et al*, 2004), but unlike other cancer systems, in the HCC model, there is transient inhibition of the proliferation pathway (p-Erk1/2), while there is persistence of the survival pathway (p-Akt), inducing cell growth inhibition and apoptosis.

Moreover, we reported that the effect of gefitinib on its downstream effectors, p-Akt and p-Erk1/2, is time-dependently reversed by Ln-5, which is expressed *de novo* in HCC but not in peritumoral or normal tissues (Giannelli *et al*, 2003), whereas no effects are observed with other ECM molecules such as Ln-1, Coll I, Coll IV, Fn, Fg and Vn. To our knowledge, this is the first evidence of an ECM molecule inhibiting the effectiveness of gefitinib, although protection from apoptosis by ECM proteins has been reported in small-cell lung carcinoma (Sethi *et al*, 1999) (Table 2).

Thus, we could hypothesise that Ln-5 expression could be a predictive factor for gefitinib response in HCC. Moreover, since Ln-5 is widely distributed in human tissues including lung and colon, our data could contribute to explain differential responsiveness to gefitinib (Haringhuizen *et al*, 2004). The mechanisms responsible for the recovery of the p-Akt form are still unknown and require further investigation; however, we can role out the possibility that Ln-5 physically interacts with gefitinib since p-EGFR is inhibited even in the presence of Ln-5. Furthermore, in our model, it is very likely that both the *α*6*β*4 and *α*3*β*1 integrins are involved in the Ln-5 signal pathway since both anti-*α*3- and anti-*α*6-blocking antibodies inhibit Ln-5-mediated survival in cytotoxicity experiments. Thus, consistently with our previous work (Giannelli *et al*, 2001), in the HCC model, it is possible that either *α*3*β*1 or *α*6*β*4 are implicated in signal transduction, mainly depending on which receptor is predominantly expressed. Therefore, these data could contribute to explain the discrepancy in the literature between the involvement of *α*3*β*1 and *α*6*β*4 in cancer aggressiveness.

In conclusion, gefitinib inhibited cell growth in all the human HCC cell lines studied. Furthermore, for the first time, we have shown that a widely distributed ECM molecule, namely Ln-5, inhibits gefitinib's ability to inhibit cell growth. For HCC patients, differential expression levels of Ln-5 may help to understand which patients might benefit from gefitinib treatment.
